# Virtual to Vital: Effectiveness of Telemedicine in Georgia

**DOI:** 10.7759/cureus.106967

**Published:** 2026-04-13

**Authors:** Akshaya Srinivasan, Angeline Abraham, Kevin Jacob, Nisrine Liyakat Nimachwala, Akshit Valsaraj Chemmancheri, Sudeeksha Sudeeksha, Yanni Premkumar Sophia, Mariam Abisonashvili

**Affiliations:** 1 Medicine, Tbilisi State Medical University, Tbilisi, GEO; 2 Faculty of Medicine, Ivane Javakhishvili Tbilisi State University, Tbilisi, GEO; 3 Family Medicine, Tbilisi State Medical University, Tbilisi, GEO; 4 Department of Family Medicine, Raymann Clinic Ltd., Tbilisi, GEO

**Keywords:** online consultation, patient compliance, patient satisfaction, physician satisfaction, physician workflow efficiency, telemedicine

## Abstract

Introduction: Telemedicine has emerged in the digital era, transforming healthcare delivery. Improving the easy accessibility, efficiency, and patient-centered care to expand healthcare access, especially for those with minimal or no access to healthcare. The issues faced in the traditional healthcare system, such as resource limitations and long waiting times, are addressed by telemedicine. This study aims to assess the effectiveness of telemedicine in Georgia using a survey-based approach employing well-established scales, such as telehealth usability and patient satisfaction questionnaires.

Methodology: A cross-sectional survey approach was used in this study to inspect telemedicine effectiveness in Georgia, focusing on patient satisfaction, compliance, physician satisfaction, and workflow efficiency. Participants comprised patients aged 18+ and healthcare providers with at least six months of telemedicine experience; those below these baselines were excluded, resulting in a sample of 80. Surveys were adapted from validated scales - PSQ-18 for patients (Cronbach’s alpha: 0.83) and the Telehealth Usability Questionnaire (TUQ) for physicians (Cronbach’s alpha: 0.5) - and distributed electronically at Raymann Hospital and via telemedicine platforms like REDMED. A 10-point Likert scale was used to assess feedback, with higher scores indicating greater satisfaction, compliance, or workflow efficiency. GraphPad Prism V.10 (La Jolla, CA, USA) was used for statistical analysis, including independent-samples t-tests to compare in-person and online consultations and Pearson correlation to assess the relationship between telemedicine use and satisfaction levels.

Results: This study revealed a numerical trend toward higher patient satisfaction with on-site consultations across both age groups, particularly among those aged 18-40, although the difference among patients aged >41 years was minimal; however, these differences were not statistically significant. Compliance followed a similar trend, with higher scores for on-site consultation; however, the differences were not statistically significant. For physicians, on-site practice was numerically associated with higher satisfaction, and while telemedicine was numerically associated with higher workflow efficiency, regardless of years of experience; however, these differences were not statistically significant. Furthermore, a strong positive correlation was observed between satisfaction and the likelihood of continuing telemedicine services for both patients (r = 0.649, p < 0.0001) and doctors (r = 0.72, p < 0.0001).

Conclusion: This study identified numerical trends suggesting notable strengths of telemedicine, including higher perceived physician work efficiency, as well as areas for improvement, and showed that older patients reported similar satisfaction levels across both consultation modes. It was observed that while on-site consultations were associated with greater patient satisfaction and compliance, telemedicine was numerically associated with higher physician workflow efficiency. A positive correlation was observed between satisfaction and sustained use of telemedicine, indicating that higher satisfaction was associated with a greater likelihood of future use. As an exploratory study, these findings should be interpreted cautiously and serve as a basis for future research. These findings suggest telemedicine may offer potential advantages worth exploring in larger studies.

## Introduction

Telemedicine promises a new phase of traditional medicine, one where the remote healthcare system is at our fingertips. In this system where technology meets healthcare consultations, telemedicine offers easy accessibility and increased efficiency while upholding the promise of patient-centered care [1]. It has evolved from simple telephone consultations to video platforms, transforming healthcare delivery worldwide.

Telemedicine is emerging in the current technologically accessible society as an adjunct to healthcare [2]. This study explores telemedicine’s efficiency and satisfaction while also linking it to its accessibility. Moreover, telemedicine addresses barriers in traditional healthcare systems, such as long wait times and resource constraints [3]. Recent studies emphasize the growing potential of integrating telemedicine into clinical practice and provide patient care comparable to that in traditional clinical settings [4]. Telemedicine can serve as an effective alternative for medical consultation, as most primary care physicians and medical specialists report it to be comparable in quality to in-person visits [5]. 

Telemedicine use has quickly grown in popularity among both the younger generation, due to its ease of access and shorter waiting periods, and the elderly, partly due to reduced transit time. This plays a significant role in overcoming barriers to accessibility for those previously excluded by traditional in-person health services. Furthermore, telemedicine adoption varies across specialties, with each requiring different levels of workload and adaptation [6]. Each of these insights highlights the transformative potential of telemedicine to improve healthcare delivery and patient experiences. Additionally, numerous online platforms facilitate telemedicine services across different countries. 

In Georgia, telemedicine platforms like REDMED and EKIMO provide easy access to patients' preferred physicians, with the option of phone or video calls [7]. These online platforms leverage technology to bridge gaps in healthcare delivery by providing telemedicine services tailored to the needs of the target population. However, existing literature on telehealth fails to address patient perspectives on telemedicine, including experiences, satisfaction, and accessibility, within the Georgian population. Moreover, the adoption and evaluation of telehealth in Georgia are constrained by limited literature, scarce resources, and a small sample size. This study directly addresses these gaps by focusing on patient satisfaction and compliance across age groups, as well as on physician satisfaction and workflow efficiency in Georgia.

The aforementioned aspects render this study unique, as it is grounded in rigorous evaluation and offers future studies to address challenges in telemedicine adoption and implementation.

The objectives of this study are to evaluate the effectiveness of telemedicine in Georgia by evaluating patient-reported satisfaction and compliance across two age groups (18-40 and 41+ years), and physician-reported satisfaction and workflow efficiency by years of telemedicine experience (less than three years and more than three years), between on-site and telemedicine services. Additionally, the study examines the correlation between satisfaction and willingness to continue telemedicine use. In Georgia, this is among the first of its kind to evaluate patient satisfaction and compliance across age groups, as well as physician satisfaction and workflow efficiency by years of experience. 

## Materials and methods

This study employed a cross-sectional survey design involving two groups (patients and physicians) to evaluate the effectiveness of telemedicine in Georgia by assessing patient satisfaction, compliance, healthcare provider satisfaction, and workflow efficiency.

The inclusion criteria for patient participants were anyone aged 18 years and older, and healthcare providers who have been using telemedicine platforms with experience of more than six months. Exclusion criteria include patients under 18 years old and healthcare providers with less than six months' experience using telemedicine platforms. A total of 80 participants were included in the sample.

Survey design and adaptation

The patient survey was based on the patient satisfaction questionnaire (PSQ-18) [8] and was modified with additional context-specific questions to ensure clarity and ease of translation, allowing for the assessment of patient satisfaction and compliance with both on-site and online consultations; this questionnaire demonstrated strong reliability with a Cronbach’s alpha score of 0.83. The questionnaire consisted of items assessing demographic characteristics, type of consultation, ease of access to healthcare services, satisfaction with provider interaction, involvement in care planning, compliance with medical recommendations, and likelihood of continuing telemedicine services (Appendix 1). Similarly, the physician survey was derived from the Telehealth Usability Questionnaire (TUQ) [9] and was adapted with context-specific questions to improve clarity and ease of translation, enabling the evaluation of physician satisfaction and workflow efficiency in both on-site and online consultation settings; this questionnaire showed limited reliability with a Cronbach’s alpha score of 0.5. Because the questionnaire was translated and contextually adapted, the scale's reliability may have been affected, and findings from the physician questionnaire should be interpreted as preliminary and exploratory. The questionnaire included items related to medical specialty, duration and frequency of telemedicine use, perceived quality of care, communication effectiveness, workload management, workflow efficiency, and willingness to continue using telemedicine services (Appendix 2).

Participant demographics

Data were collected from the following specialties: family medicine, radiology, endocrinology, dermatology, and pediatrics. Family medicine doctors comprised the majority of physicians, reflecting their pivotal role in healthcare delivery.

Data collection and distribution

The surveys were distributed electronically via Google Forms to ensure ease of access and participation among doctors and patients at Raymann Hospital in Tbilisi, Georgia, and to all physicians registered on telemedicine platforms in Georgia, such as REDMED. This approach ensures broad reach and increased participation among physicians and patients actively using telemedicine services. Because the surveys were distributed electronically, the exact response rate could not be calculated.

Participants rated their experience on a 10-point Likert scale [10], with higher scores indicating greater satisfaction, compliance, or workflow efficiency.

Statistical analysis

The collected data were analyzed using GraphPad Prism V.10 software (La Jolla, CA, USA) [11], generating statistical outputs and graphs to support this study. Statistical methods include independent-samples t-tests, which were used to compare patient satisfaction and compliance, as well as physician satisfaction and workflow efficiency, between on-site and online consultations.

In addition, Pearson correlation was used to examine the relationship between telemedicine practices and the likelihood of continuing telemedicine services.

Independent t-tests were used to compare the outcomes between the groups. Given the modest sample size and exploratory design, the findings should be considered hypothesis-generating rather than confirmatory.

Ethical considerations

This study adheres to ethical research standards and obtained approval from the Institutional Review Board (IRB). Informed consent was obtained from all participants before data collection, ensuring confidentiality of the responses and voluntary participation. No personal or identifiable data were collected during this study.

## Results

Of the 80 responses received, 40 were from patients and 40 from physicians.

Demographic data

**Figure 1:**
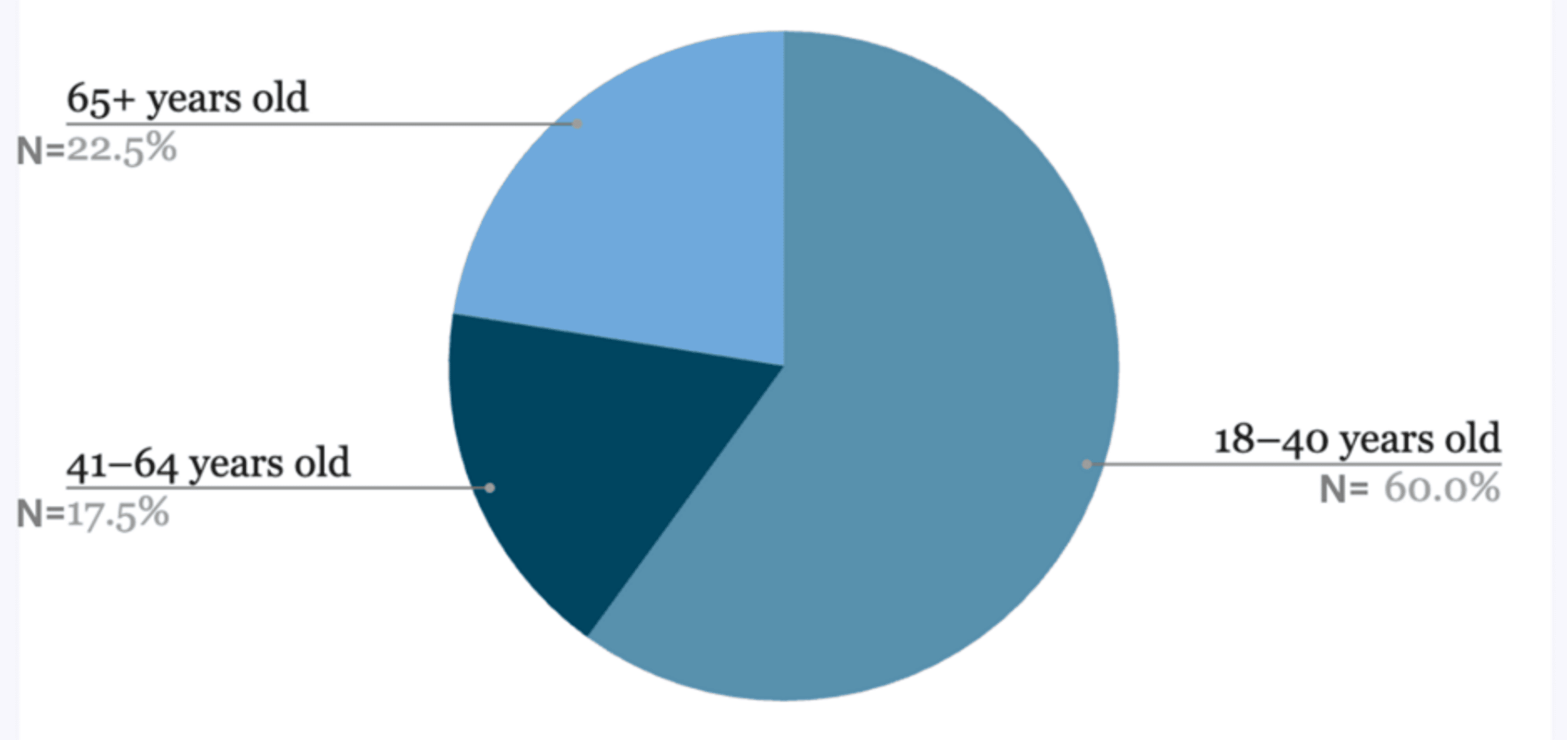
Age distribution of the patients The pie chart illustrates the age distribution of patients in the study. Sixty percent of patients were aged 18 to 40 years, followed by 22.5% aged 65 years and older, and 17.5% aged 41 to 64 years. Percentages represent the proportion of patients within each age group.

**Figure 2:**
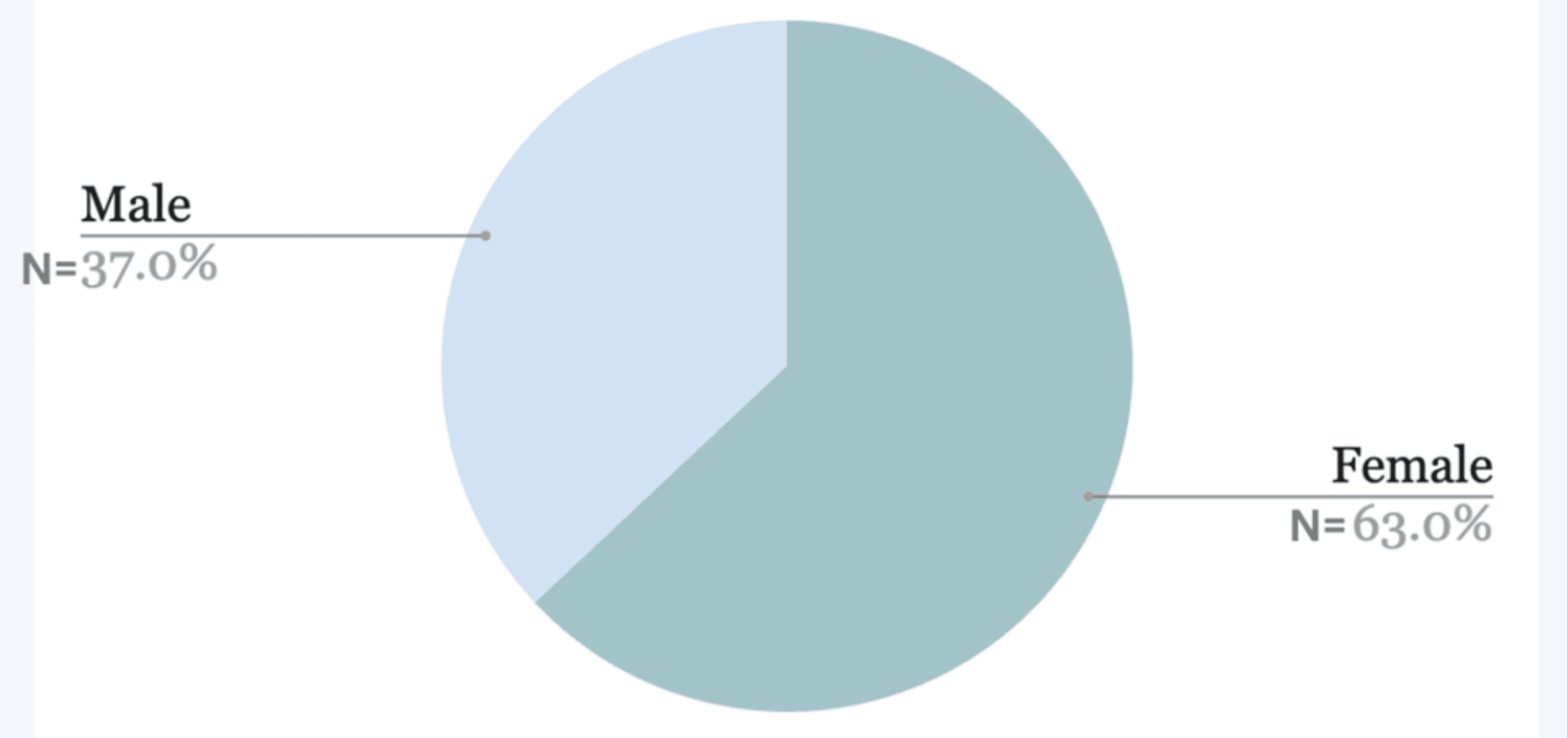
Gender distribution of the patients Females represent 63%, which is majority of the patients, and male patients account for 37%.

This study showed that 60% of the patient population was aged between 18 and 40, followed by 22.5% of the patient population aged 65 and above, and lastly, 17.5% were aged 41-64 years from the same sample pool (Figure 1). Sixty-three percent of this population were females, and the rest were males (37%) (Figure 2).

**Figure 3:**
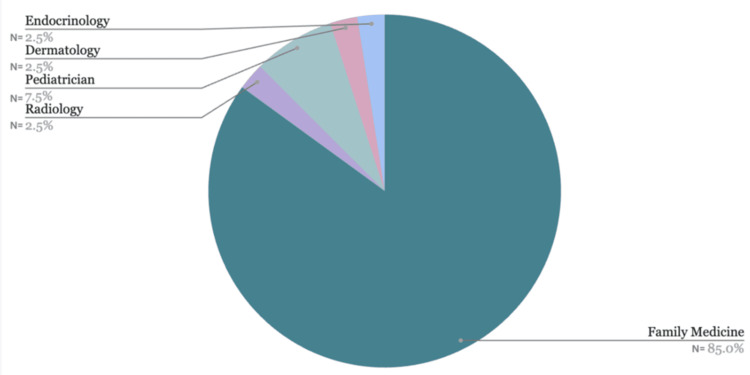
The distribution of physician specialties The majority of physicians specialize in Family Medicine, accounting for 85% of the total. Other specialties include Pediatrics (7.5%), Endocrinology (2.5%), Dermatology (2.5%), and Radiology (2.5%). The chart highlights the overwhelming dominance of Family Medicine among physician specialties.

The demographics of the doctors' specialties showed that the majority were Family Doctors, at 85%, followed by Pediatricians (7.5%), with the remainder comprising Endocrinologists, Dermatologists, and Radiologists, each at 2.5% (Figure 3). 

Health conditions

**Figure 4:**
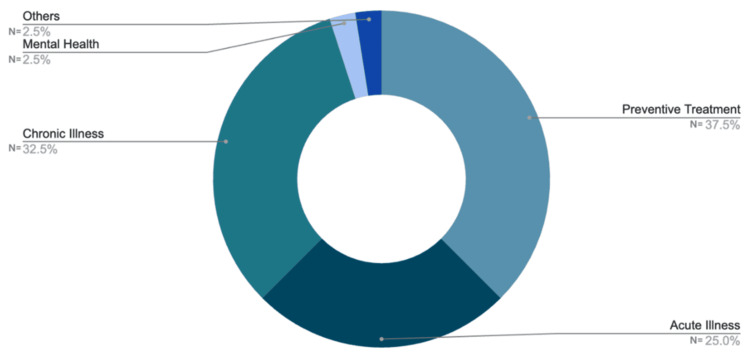
Medical conditions distribution The donut chart illustrates the distribution of medical conditions among patients. Preventive treatment comprises of the majority (37.5%), followed by chronic illness (32.5%) and acute illness (25%). Mental health and other conditions each represent 2.5%. Percentages indicate the proportion of cases in each category.

Preventive treatment accounts for 37.5% of cases. Management of chronic diseases such as diabetes and hypertension accounted for 32.5% of cases, while acute illnesses accounted for 25%. Mental health concerns, followed by other consultations such as prescription-related issues, accounted for 2.5% of telemedicine usage (Figure 4).

Patient satisfaction on-site versus telemedicine

**Figure 5:**
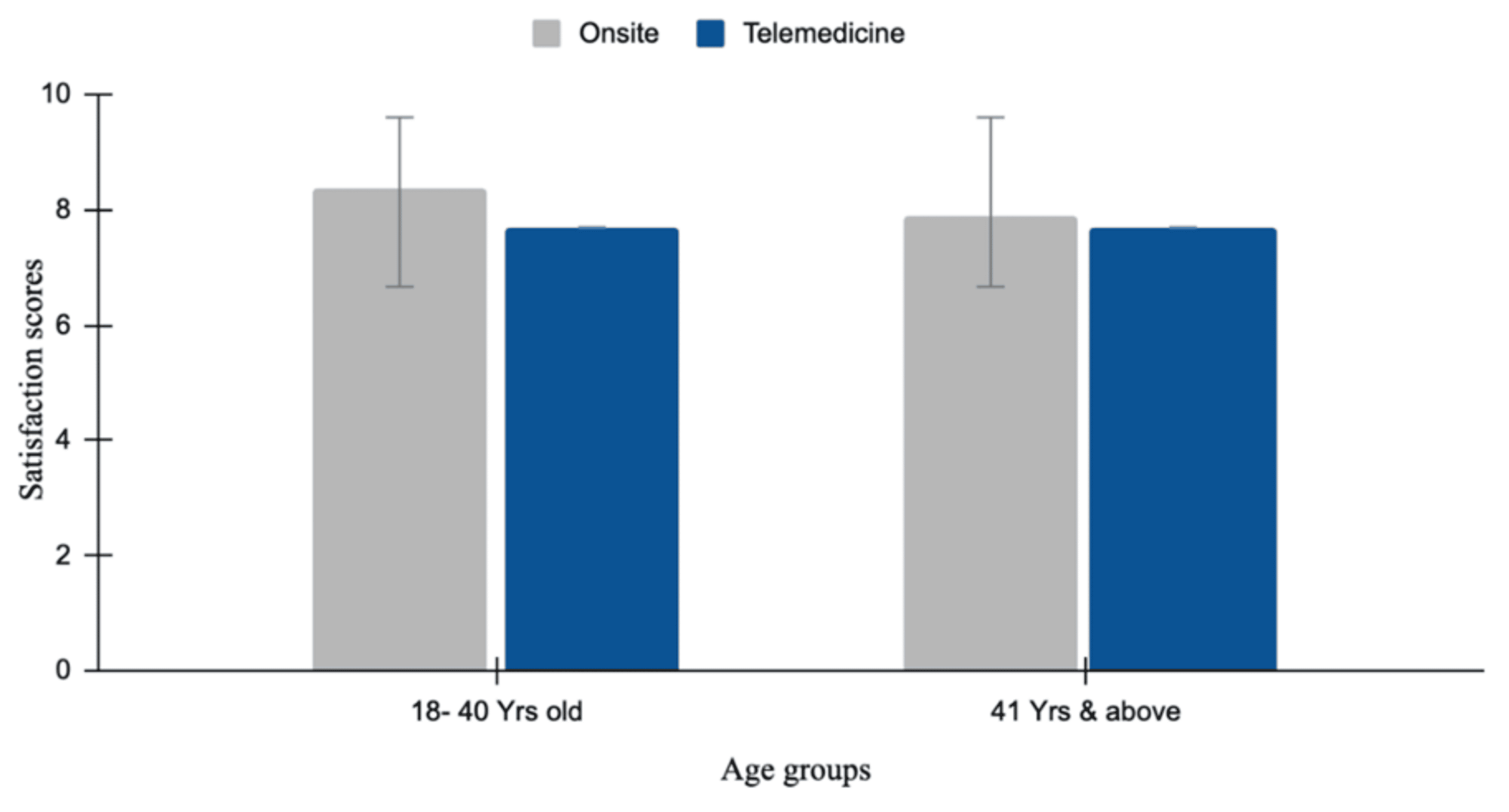
Patient satisfaction on-site versus telemedicine The bar chart compares patient satisfaction scores between on-site consultations and telemedicine across two age groups: 18–40 years and 41 years and above. For the 18–40 years group, the mean satisfaction score for on-site consultations (grey) is 8.38, compared to 7.7 for telemedicine (blue). Similarly, for the 41 years and above group, the mean satisfaction score for on-site consultations (grey) is 7.9, compared to 7.7 for telemedicine (blue). Error bars represent the standard deviation.

Of the 40 responses, satisfaction levels between the 18-40 and 41+ year age groups for both online and on-site consultations were similar. The 18-40-year age group numerically reported higher satisfaction with on-site practices (mean 8.38) than the telemedicine group (mean 7.7). The 41+ age group shows slightly lower satisfaction levels for on-site and online consultations, with mean values of 7.9 and 7.7, respectively. For on-site consultations, the independent-samples t-test revealed a statistically insignificant difference in satisfaction scores (p=0.39), with a mean difference of -0.4542 (95% CI: -1.522 to 0.6140). Online consultations have also revealed a statistically insignificant difference in satisfaction levels (p=0.99), with a mean difference of -0.004 (95% CI: -1.108 to 1.09) (Figure 5).

Patient compliance on-site versus telemedicine

**Figure 6:**
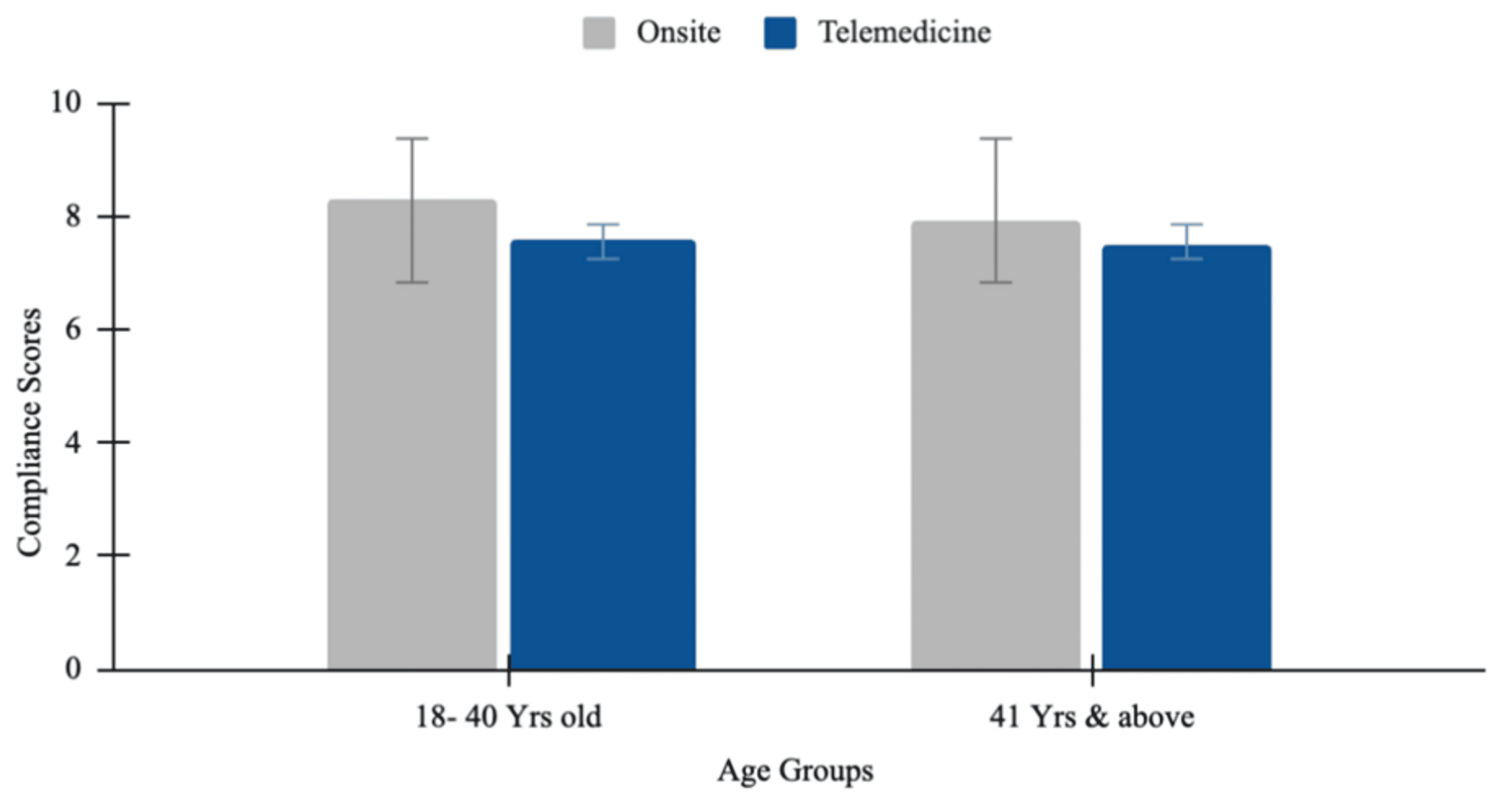
Patient compliance on-site versus telemedicine This bar chart compares patient compliance scores between on-site and telemedicine visits across two age groups: 18 to 40 years old and 41 years and above. Compliance scores are higher for on-site visits compared to telemedicine in both age groups. For the 18 to 40 years group, the mean compliance scores are 8.3 (on-site, grey) and 7.6 (telemedicine, blue). For those 41 years and above, the mean scores are 7.9 (on-site, grey) and 7.5 (telemedicine, blue). The error bars represent standard deviations.

Compliance levels across both age groups showed a similar trend, reporting slightly higher compliance with on-site practices than with online consultations, with marginal differences. The 18-40 age group reported higher compliance with on-site consultations, with a mean of 8.3, compared with 7.6 for online consultations. The 41+ age group similarly showed higher compliance with on-site consultations, with a mean of 7.9, compared with telemedicine consultations, which had a mean of 7.5. The independent-samples t-test was statistically insignificant for on-site consultations (p= 0.49), with the mean difference of -0.38 (95% CI: -1.49 to 0.73). For online consultations, the compliance levels were also statistically insignificant (p=0.7), with a mean difference of 0.18 (95% CI: -0.811 to 1.182) (Figure 6).

Physician satisfaction on-site versus telemedicine

**Figure 7:**
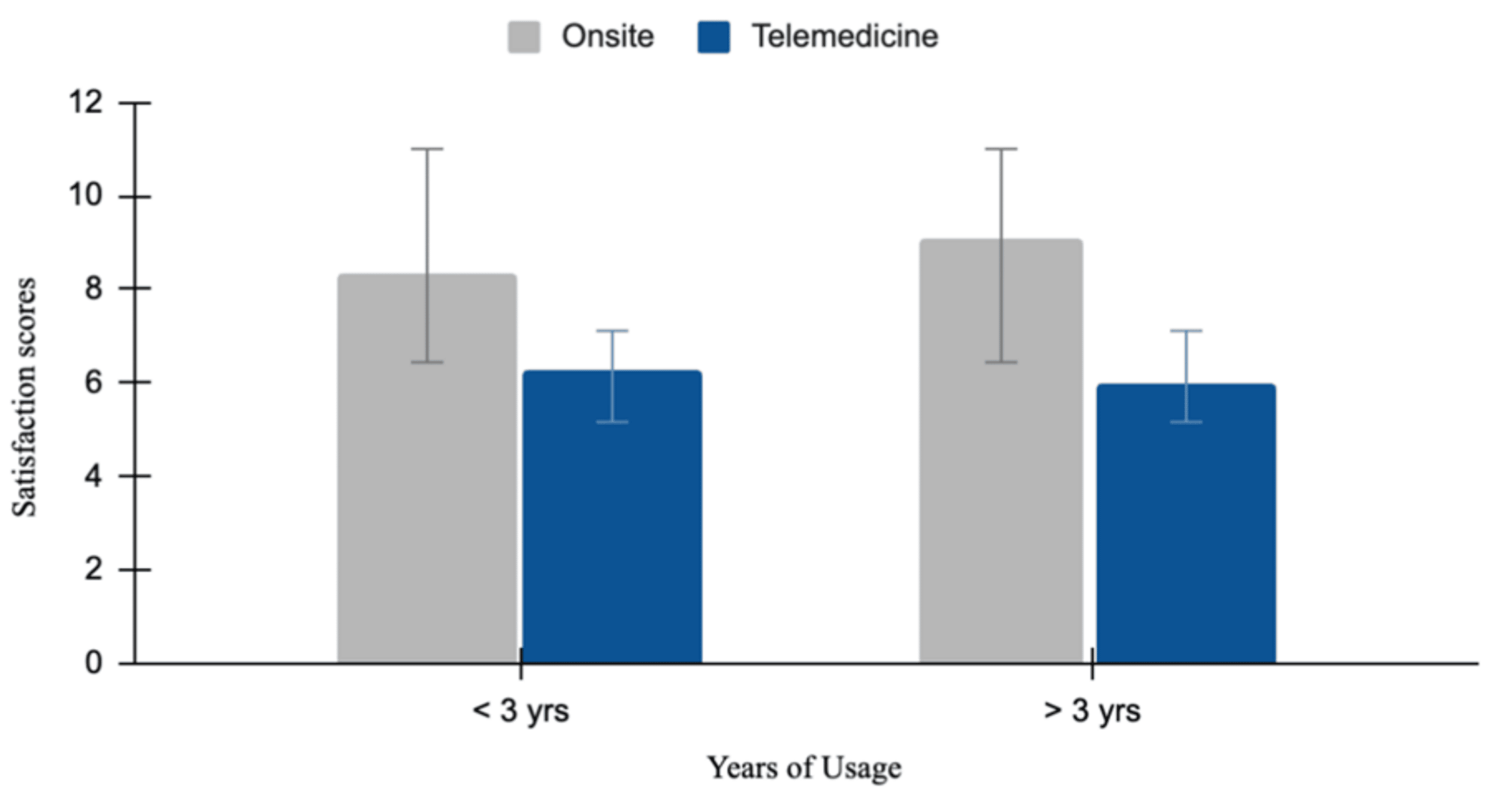
Physician satisfaction on-site versus telemedicine This bar chart compares physician satisfaction scores between on-site and telemedicine visits based on years of usage (less than three years and more than three years). For physicians with less than three years of usage, the mean satisfaction scores are 8.35 (on-site, grey) and 6.3 (telemedicine, blue). For those with more than three years of usage, the mean scores are 9.1 (on-site, grey) and 5.98 (telemedicine, blue). Error bars represent standard deviations.

Physician satisfaction reported higher satisfaction levels for on-site consultations with a mean value of 8.35 (less than three years of experience) and 9.1 (more than three years of experience), in comparison to online consultations with mean values of 6.3 (less than three years of experience) and 5.98 (more than three years of experience). The independent-samples t-test for on-site consultations was not statistically significant (p = 0.24), with a mean difference of 0.78 (95% CI: -0.61 to 2.18). For online consultations, physician satisfaction levels also show a statistically non-significant result (p = 0.24), with a mean difference of -0.36 (95% CI: -2.3 to 1.57). These results show a numerical trend toward higher physician satisfaction with on-site consultations, though these differences were not statistically significant (Figure 7).

Physician work efficiency on-site versus telemedicine

**Figure 8:**
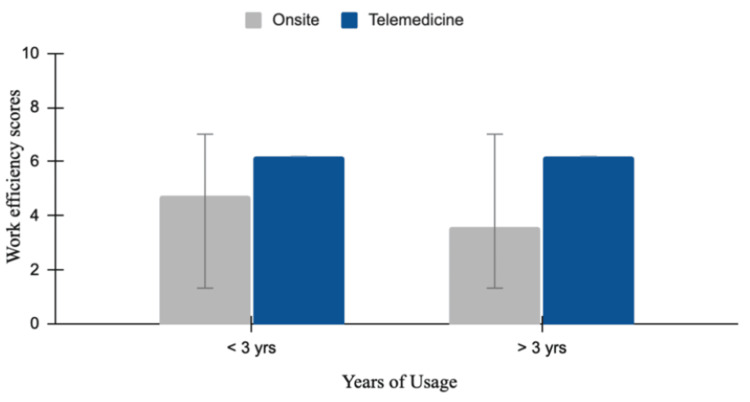
Physician work efficiency on-site versus telemedicine The bar graph shows a comparison between physicians' work efficiency scores between on-site and telemedicine consultations across two groups based on years of telemedicine usage: less than three years and more than three years. Efficiency was measured using a 10-point Likert scale, with higher scores indicating greater efficiency. For physicians with less than three years of telemedicine usage, the mean scores are 4.75 (on-site, grey) and 6.2 (telemedicine, blue). Error bars represent the standard deviation (SD) of the mean.

Work efficiency levels were higher with online consultation, with mean values of 6.2 for both less than three years and more than three years of usage. However, efficiency levels were lower with on-site practices, with mean values of 4.75 (less than three years of usage) and 3.6 (more than three years of usage). For on-site work efficiency, the difference was statistically insignificant (p = 0.15), with a mean difference of -1.08 (95% CI: -2.6 to 0.4. For work efficiency in the online mode of telemedicine, the levels were low and showed a statistically insignificant difference (p = 0.84), with a mean difference of 0.133 (95% CI: -1.28 to 1.54). These findings suggest a numerical trend towards higher workflow efficiency with telemedicine; however, the differences between groups were not statistically significant (Figure 8).

Patient satisfaction versus telemedicine services

**Figure 9:**
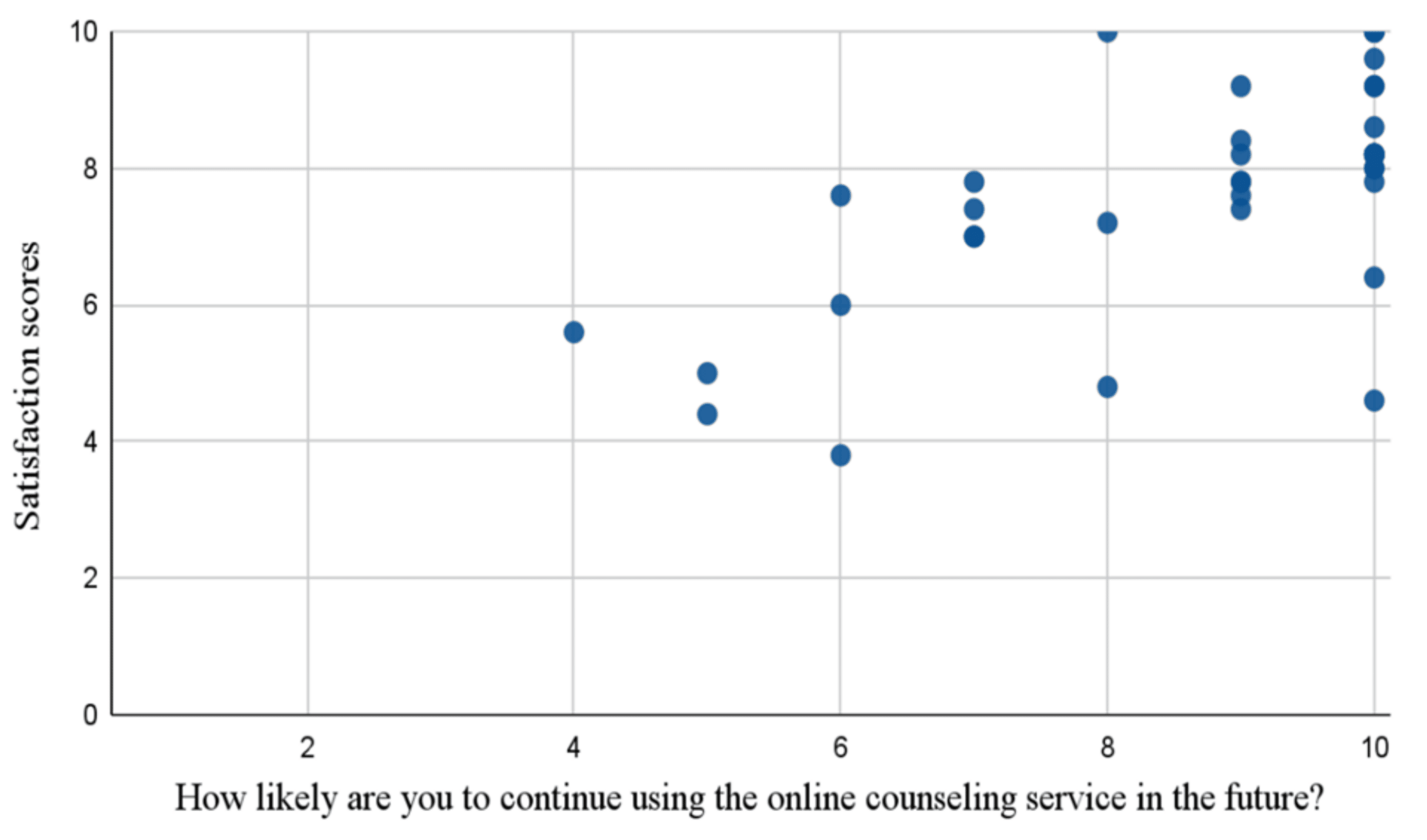
Patient satisfaction versus telemedicine services This scatter plot illustrates the relationship between patient satisfaction scores (y-axis) and their likelihood of continuing to use telemedicine services in the future (x-axis). Each point represents an individual response, with satisfaction scores ranging from 0 to 10 and likelihood scores from 1 to 10. The data indicates a positive trend, suggesting that higher patient satisfaction is associated with a greater likelihood of continuing telemedicine services.

A positive relationship was found between patient satisfaction scores and their likelihood of continuing telemedicine services, with a correlation coefficient (r) of +0.649, indicating a moderately strong relationship. The 95% confidence interval (CI) ranges from 0.41 to 0.80, confirming this relationship. The approximate p-value is <0.0001, indicating statistical significance. Patients with higher satisfaction scores (7-10) report greater likelihood scores (8-10) while lower satisfaction scores (2-5) correspond to lower likelihood scores (1-4). Mid-range satisfaction scores (4-8) correspond to moderate likelihood scores of (5-7). Overall, this trend suggests that as the patient’s satisfaction scores increase, so do their likelihood scores, emphasizing satisfaction as a strong predictor of willingness to continue telemedicine services (Figure 9). As both variables were measured concurrently via self-report, this correlation reflects a concurrent association rather than a predictive or causal relationship. 

Physician satisfaction versus telemedicine services

**Figure 10:**
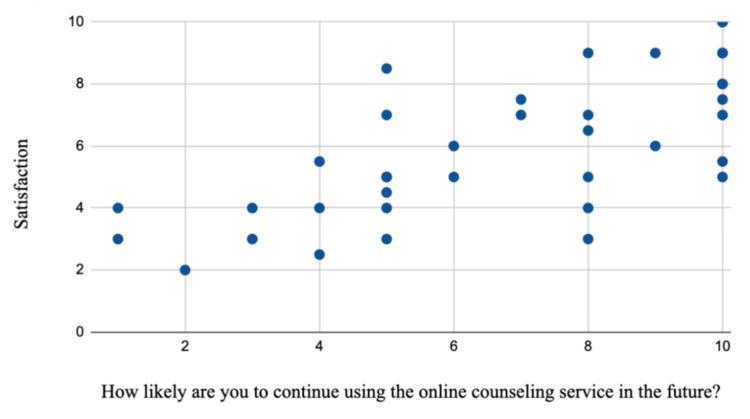
Physician satisfaction versus telemedicine services This scatter plot illustrates the relationship between physician satisfaction scores (y-axis) and their likelihood of continuing to use telemedicine services in the future (x-axis). Each point represents an individual response, with satisfaction scores ranging from 0 to 10 and likelihood scores from 1 to 10. The data indicates a positive trend, suggesting that higher physician satisfaction is associated with a greater likelihood of continuing telemedicine services.

A similarly positive correlation was found between physician satisfaction and the likelihood of continuing telemedicine services, as evidenced by a correlation coefficient (r) of +0.72. The 95% confidence interval ranges from 0.52 to 0.84, confirming the correlation. The approximate p-value is < 0.0001, which indicates statistical significance. Higher satisfaction scores (7-10) were predominantly associated with higher likelihood scores (8-10), while lower satisfaction scores (2-4) corresponded to lower likelihood scores (1-4). Mid-range satisfaction scores (3-8) correspond to moderate likelihood scores of (5-7). These findings demonstrate that satisfaction significantly influences doctors' willingness to continue telemedicine services (Figure 10). As both variables were measured concurrently via self-report, this correlation reflects a concurrent association rather than a predictive or causal relationship. 

## Discussion

This study explored the perceived satisfaction, compliance, and workflow efficiency across on-site and telemedicine consultations in Georgia. Although no statistically significant differences were observed in the analysis, several numerical trends were identified that could inform future research [12].

The results show no statistically significant differences in satisfaction and compliance across all age groups between the on-site and online consultation platforms. However, patients aged 18 to 40 years reported a trend toward higher satisfaction and compliance with on-site consultations, reflecting a preference for in-person interactions for immediate care. In contrast, the older population (41 years and above) showed almost equal levels of satisfaction and compliance with both online and off-site consultations. This may reflect their appreciation for the convenience and reduced effort in meeting travel demands that telemedicine facilitates. Regarding the health conditions for which telemedicine was commonly used, younger patients primarily used it for preventive care, such as routine health checkups, while older patients used it more frequently to manage chronic conditions like hypertension and diabetes. Preventive treatment, such as routine health check-ups, used telemedicine the most, accounting for 37.5% of cases. This could suggest that patients accept telemedicine for general health monitoring, as it is more accessible and convenient. Management of chronic diseases like diabetes and hypertension accounted for 32.5% of cases, while acute illnesses such as the common cold, flu, and other acute infections accounted for 25%. These findings suggest that telemedicine may support the management of long-term conditions by reducing the frequency of visits and, for acute illnesses, can serve as an adjunct to urgent care for minor conditions. In contrast, mental health concerns like depression and anxiety, followed by prescription-related consultations, comprised 2.5% of telemedicine usage, respectively. The lower adoption rates may suggest a preference for in-person care and accessibility that is needed in psychiatric conditions. These statistics highlight the predominant reliance on telemedicine for routine and chronic care.

Despite these benefits, on-site consultations remained the preferred choice for most patients, as technical issues, such as internet connectivity problems, caused miscommunication between patients and physicians and were a significant barrier to telemedicine use. To address this problem, a non-randomized controlled trial conducted in 2023 introduced the teach-back method in telemedicine to improve communication during telehealth consultations. This strategy involves healthcare professionals explaining medical information to patients and then asking them to repeat it in their own words to assess their understanding [13].

In a 2022 study conducted in Georgia, healthcare professionals expressed overall satisfaction with telemedicine, with 41% rating it 4 out of 5. The majority of surveyed professionals expressed a positive attitude towards telemedicine and were motivated to work on it consistently. This study perceived telemedicine as having immense potential and promise for the future [14]. This calls for improved telemedicine platforms like REDMED and EKIMO, as well as better digital literacy. However, it also shows that physicians reported higher satisfaction with on-site practices, suggesting that traditional in-person care continues to offer greater professional fulfillment, most likely due to better patient-centric interactions and fewer technological barriers.

The work efficiency among physicians was reported to be higher with telemedicine practices, regardless of years of experience; however, these differences were not statistically significant. This may be related to schedule flexibility and better management of patient loads, while physicians reported no perceived compromise in quality of care, although objective clinical outcomes were not assessed. Although the differences between on-site and telemedicine practices in terms of physician satisfaction and work efficiency were not statistically significant, they may stem from technological challenges associated with telemedicine and scheduling errors. Despite these challenges, telemedicine services, over time, highlights its potential to become a more viable alternative as providers gain familiarity with the platform. These observations suggest that increased telemedicine use could improve work efficiency, optimize time utilization, enhance productivity, and reduce administrative load, though larger studies with objective measures of productivity are needed to confirm this.

Both patient and physician satisfaction showed moderate-to-strong positive correlations with the likelihood of continuing telemedicine services, with correlation coefficients of +0.649 and +0.72, respectively. These findings emphasize satisfaction as a critical determinant of telemedicine’s long-term usage. Patients with higher satisfaction were more likely to continue using telemedicine, highlighting the need to reduce technical errors and improve the platform's user-friendliness to enhance their experience.

This study has several limitations. First, the relatively small sample size (n = 80) limits the generalizability of the findings and may reduce the study's statistical power. Second, participants were recruited via convenience sampling, and the response rate could not be calculated, which introduces potential selection bias. Third, the physician questionnaire showed that low internal consistency (Cronbach's alpha = 0.5) may compromise the reliability of measurements of workflow efficiency and usability. Additionally, the cross-sectional and self-reported nature of the survey limits the ability to infer causal relationships between telemedicine use and satisfaction or efficiency outcomes. Finally, the use of Likert-scale responses may introduce subjectivity in participant reporting. However, despite digital limitations, strong communication skills, an understanding of patient concerns, and comprehension of treatment are essential for successful interactions. Thus, a strong patient-physician relationship is vital to the continued success of telemedicine practices [15].

The findings of this study align with the existing literature, suggesting that telemedicine services may help to bridge the gaps in current healthcare systems. Nonetheless, the identified challenges, such as lower satisfaction and efficiency levels in telemedicine compared to on-site practices, highlight the need for targeted interventions. These include addressing technological accessibility by exploring the impact of telemedicine in offline settings and low-data platforms on both patient and physician engagement. Further literature is also needed to evaluate how providing comprehensive training and support for patients and providers can enhance technological literacy and improve compliance with telemedicine tools. A customized approach should also be examined, as tailoring telemedicine services to different demographics will affect their effectiveness and satisfaction. Finally, future studies should adopt a specialty-focused approach, exploring areas like tele-cardiology (AI-driven remote cardiac monitoring) [16], tele-oncology (virtual chemotherapy supervision and AI-assisted cancer detection) [17], tele-neurology (remote stroke care and seizure monitoring), tele-psychiatry (AI-driven mental health screening and VR therapy) [18], and tele-endocrinology (wearable hormone and glucose monitoring) [19], to better understand the role and importance of telemedicine in various specialty-focused areas.

Moving forward, these initiatives can address barriers that lead to dissatisfaction and reduced compliance, thereby enhancing telemedicine use and acceptance, integrating it into clinical practice, and possibly improving the user experience for physicians.

## Conclusions

This exploratory study identified patterns in perceived satisfaction, compliance, and workflow efficiency across on-site and telemedicine consultations in Georgia. This study highlights the notable strengths of telemedicine, including comparable satisfaction among older patients and increased physician work efficiency, while identifying areas for improvement. Satisfaction and compliance are studied for patients, while satisfaction and workflow efficiency are studied for physicians. It was observed that while on-site consultations were numerically associated with greater patient satisfaction and compliance, telemedicine enhanced physicians' workflow efficiency; however, none of these differences were statistically significant and should be considered directional trends rather than confirmed comparative advantages. A positive correlation was observed between satisfaction and continued telemedicine use, indicating that greater satisfaction is associated with a higher likelihood of future use, though both variables were self-reported concurrently, limiting causal interpretation. Future research must explore additional factors such as patient outcomes, a specialty-focused approach, and increased accessibility in offline settings. Large-scale, rigorous studies using validated tools, outcome measures, and well-defined comparative designs are required to underscore these preliminary findings.

## Appendices

Appendix 1 

**Table 1:** Patient questionnaire Modified from the Patient Satisfaction Questionnaire (PSQ-18) [8]

Qns No.	Patient survey questionnaires
1	What is your age?
2	What is your gender?
3	For what health condition or other reason are you using the online consultation service?
4	What type of consultation?
5	How easy is it for you to visit the clinic for your regular health checks?
6	How satisfied are you with the time your health care provider spends with you during clinic visits?
7	How likely are you to continue with regular health checks through clinic visits?
8	How involved are you in developing your health improvement plan during your clinic visit?
9	How often do you follow your healthcare provider's recommendations during clinic visits?
10	How easy is the online consultation service to check your health?
11	How easy are the instructions for using the online counseling service?
12	How satisfied are you with the time allocated to you by the medical staff during online consultations?
13	How comfortable and convenient is using your online consultation service compared to an on-site visit?
14	How likely are you to continue using the online counseling service in the future?
15	How often do you follow the recommendations of the medical staff after checking online?
16	How involved do you feel in developing your health improvement plan during the online consultation?
17	Have you experienced any discomfort during online counseling?

Appendix 2 

**Table 2:** Physician Questionnaire Modified from the Telehealth Usability Questionnaire (TUQ) [9]

Qns No.	Physician survey questionnaires
1	What is your specialty?
2	Approximately how many online consultations have you done?
3	How long have you been using the online counseling service?
4	What types of online counseling services do you use?
5	On-site visits do little to help me manage my work time.
6	How would you rate the quality of care received from an on-site consultation compared to an online consultation?
7	I feel that on-site consultation allows me to communicate more effectively with my patients.
8	On- site visit doesn't help me handle my workload more efficiently
9	⁠Compared to telemedicine, on-site visits do not simplify patient monitoring.
10	I believe that the online consultation system is easy to use.
11	I am satisfied with the quality of help I provide to my patients through online consultation.
12	Online counseling helps me manage my workload more efficiently.
13	I think online consultation allows me to communicate effectively with my patients.
14	Telemedicine simplifies patient monitoring.
15	What challenges or inconveniences did you face when using the online counseling service?
16	What details would you like to correct in the online consultation system?
17	How likely are you to continue with online consultations?
